# A SARS-CoV-2 RBD vaccine fused to the chemokine MIP-3α elicits sustained murine antibody responses over 12 months and enhanced lung T-cell responses

**DOI:** 10.3389/fimmu.2024.1292059

**Published:** 2024-02-02

**Authors:** James Tristan Gordy, Yinan Hui, Courtney Schill, Tianyin Wang, Fengyixin Chen, Kaitlyn Fessler, Jacob Meza, Yangchen Li, Alannah D. Taylor, Rowan E. Bates, Petros C. Karakousis, Andrew Pekosz, Jaiprasath Sachithanandham, Maggie Li, Styliani Karanika, Richard B. Markham

**Affiliations:** ^1^ W. Harry Feinstone Department of Molecular Microbiology and Immunology, Bloomberg School of Public Health, Johns Hopkins University, Baltimore, MD, United States; ^2^ Division of Infectious Diseases, Department of Medicine, Johns Hopkins School of Medicine, Baltimore, MD, United States

**Keywords:** SARS-CoV-2, vaccine, dendritic cell, MIP-3α, intranasal (IN), T-cell response, antibody, neutralizing antibody

## Abstract

**Background:**

Previous studies have demonstrated enhanced efficacy of vaccine formulations that incorporate the chemokine macrophage inflammatory protein 3α (MIP-3α) to direct vaccine antigens to immature dendritic cells. To address the reduction in vaccine efficacy associated with a mutation in severe acute respiratory syndrome coronavirus 2 (SARS-CoV-2) mutants, we have examined the ability of receptor-binding domain vaccines incorporating MIP-3α to sustain higher concentrations of antibody when administered intramuscularly (IM) and to more effectively elicit lung T-cell responses when administered intranasally (IN).

**Methods:**

BALB/c mice aged 6–8 weeks were immunized intramuscularly or intranasally with DNA vaccine constructs consisting of the SARS-CoV-2 receptor-binding domain alone or fused to the chemokine MIP-3α. In a small-scale (*n* = 3/group) experiment, mice immunized IM with electroporation were followed up for serum antibody concentrations over a period of 1 year and for bronchoalveolar antibody levels at the termination of the study. Following IN immunization with unencapsulated plasmid DNA (*n* = 6/group), mice were evaluated at 11 weeks for serum antibody concentrations, quantities of T cells in the lungs, and IFN-γ- and TNF-α-expressing antigen-specific T cells in the lungs and spleen.

**Results:**

At 12 months postprimary vaccination, recipients of the IM vaccine incorporating MIP-3α had significantly, approximately threefold, higher serum antibody concentrations than recipients of the vaccine not incorporating MIP-3α. The area-under-the-curve analyses of the 12-month observation interval demonstrated significantly greater antibody concentrations over time in recipients of the MIP-3α vaccine formulation. At 12 months postprimary immunization, only recipients of the fusion vaccine had concentrations of serum-neutralizing activity deemed to be effective. After intranasal immunization, only recipients of the MIP-3α vaccine formulations developed T-cell responses in the lungs significantly above those of PBS controls. Low levels of serum antibody responses were obtained following IN immunization.

**Conclusion:**

Although requiring separate IM and IN immunizations for optimal immunization, incorporating MIP-3α in a SARS-CoV-2 vaccine construct demonstrated the potential of a stable and easily produced vaccine formulation to provide the extended antibody and T-cell responses that may be required for protection in the setting of emerging SARS-CoV-2 variants. Without electroporation, simple, uncoated plasmid DNA incorporating MIP-3α administered intranasally elicited lung T-cell responses.

## Introduction

The severe acute respiratory syndrome coronavirus 2 (SARS-CoV-2) pandemic has provided yet one more example of how the mutability of RNA viruses hinders efforts to develop effective immunoprophylactic strategies. Emerging variants have been uniformly less susceptible to neutralizing antibodies elicited by exposure to earlier vaccines or prior infection (1–9). Resistance of new variants to pre-existing neutralizing antibodies is not absolute but varies with both the extent of mutation in vaccine-targeted proteins and the concentration of neutralizing antibodies targeting previous viral variants (4–6, 10). While repeated immunization targeting variants of the SARS-CoV-2 has proceeded as a strategy for addressing waning protective immunity, there has been sustained resistance to repeated immunization, as evidenced by surveys of current attitudes toward vaccination (11) and the small proportion of Americans who received the most recent vaccine booster (12).

Although declining antibody concentrations increase the risk of acquiring SARS-CoV-2 infection, evidence from clinical and non-human primate studies has indicated that the currently available vaccines may elicit T-cell-mediated immune responses that are less susceptible than neutralizing antibody responses to the immune escape associated with SARS-CoV-2 variants (12–15). These T-cell responses may at least partially explain how vaccines that elicit relatively short-lived neutralizing antibodies can still attenuate disease severity without preventing infection (16).

These findings indicate that vaccine development efforts to counter the ongoing emergence of variants should include the development of vaccines that elicit both more sustained antibody concentrations and T-cell responses with activity at the site of infection. Unlike the situation for circulating neutralizing antibodies elicited by immunization or previous infection that are readily accessible at the time of pathogen exposure, T-cell immunity is most effective if pathogen-specific T cells pre-exist within the targeted organ (16–19). To counter respiratory pathogens, multiple studies have indicated that intranasal (IN) immunization is more effective than systemic immunization at eliciting T-cell responses in the lungs (20–22).

In murine vaccine model systems analyzing protective or therapeutic efficacy against malaria, tuberculosis, and melanoma, we have studied the impact of fusing vaccine antigen to the chemokine macrophage inflammatory protein 3α (MIP-3α), also known as chemokine (C–C motif) ligand 20 (CCL20). Both human and mouse MIP-3α are able to bind mouse (23) C–C motif chemokine receptor 6 (CCR6), a member of the G protein-coupled receptor family found on immature but not mature dendritic cells (DC). Targeting immature DC (iDC) distinguishes this vaccine construct from other dendritic cell-targeting vaccines, which engage receptors expressed by more differentiated DC (24–29) to enhance vaccine efficacy. Employment of this vaccine platform has generated greater and, in some model systems, more sustained responses than those observed when the vaccine antigen is not fused to the chemokine (22, 23, 30–32). While MIP-3α serves as a chemoattractant for iDC (33–38), previous studies have demonstrated the requirement of fusing the vaccine antigen to chemokine to achieve maximum efficacy. In our recent study in a mouse challenge model using a therapeutic vaccine targeting the *Mycobacterium tuberculosis* stringent response, we found that IN immunization elicited significantly greater antibacterial activity than intramuscular (IM) immunization, and that IN vaccination with the MIP-3α-fused vaccine was also significantly more effective than immunization with vaccine antigen alone (22). Optimal immune responses were observed with a DNA vaccine construct that required no encapsulation of the DNA or use of adjuvant to achieve the observed therapeutic efficacy.

In the current study, we have examined in a mouse model the comparative immunogenicity of a DNA vaccine incorporating the receptor-binding domain (RBD) of the SARS-CoV-2 spike protein with or without fusion to human MIP-3α. Our results indicate that, over a year of observation, the fusion vaccine elicited and sustained significantly higher antibody concentrations compared to the vaccine incorporating RBD alone. Of particular note was the persistence in the recipients of the fusion vaccine of neutralizing antibody responses for at least 4 months after they were no longer detectable in the recipients of the vaccine only encoding RBD. Furthermore, we found that IN immunization yielded significantly greater T-cell responses in the lung than those elicited by IM immunization, but only when RBD was fused to MIP-3α.

## Methods

### Vaccine plasmid construction and verification

pUC57 plasmid containing DNA encoding codon-optimized RBD (spike amino acids 319–545 of Wuhan-Hu-1 isolate) was purchased from GenScript (Piscataway, NJ, USA). DNA was extracted and ligated into a previously generated pSecTag2b plasmid by *Hind*III and *Bam*HI to generate RBD alone and also by *Kas*I and *Bam*HI to generate MIP-3α-RBD (restriction enzymes from Thermo Fisher, Waltham, MA, USA) (22). Proper insertion was confirmed by agarose gel electrophoresis and sequencing, and the expression of target genes was confirmed by immunoblots probed by anti-C-myc (BioLegend, San Diego, CA, USA) of cell lysates and supernatants following transfection of HEK293T cells (American Type Culture Collection, Manassas, VA, USA) utilizing the Trans-IT 293 transfection system (Mirus Bio, Madison, WI, USA) (**Supplementary Figure S1**). In brief, lysates were prepped using 10× Cell Lysis Buffer (Cell Signaling Tech., Danvers, MA, USA) according to manufacturer protocol, protein amount was normalized utilizing Bradford Assay (Oz Biosciences, San Diego, CA, USA), separated on precast TGX Gels (Bio-Rad, Hercules, CA, USA), transferred to nitrocellulose membranes (Bio-Rad), blocked with milk solution, probed with anti-C-myc for 2 h to overnight at 1:1,000 dilution, washed, probed with AP-conjugated goat antimouse antibody (Jackson ImmunoResearch Laboratories, Inc., West Grove, PA, USA) at 1:1,000 dilution for 1 h, washed, and visualized with NBT-BCIP reagent (Sigma Aldrich, St. Louis, MO, USA). Vaccination plasmids were selected with ampicillin (100 mg/mL), and Qiagen^®^ Endo-Free^®^ Plasmid Series kits were used to extract the ligation product from DH5-α *Escherichia coli* (Invitrogen™, Thermo Fisher Scientific, Waltham, MA, USA). Plasmid DNA was diluted with endotoxin-free 1× PBS. Nanodrop spectrophotometry, agarose gel electrophoresis, and insert sequencing (JHSSF) were used to test the concentration, purity, and correctness of the extracted DNA.

### Mice

BALB/c mice aged 6 to 8 weeks old were purchased from Charles River Laboratories Inc., Wilmington, MA, USA. The long-term experiment utilized all female mice. The intranasal experiment utilized a parallel distribution of male and female mice. All mice were kept in a pathogen-free micro-isolation facility at Johns Hopkins in accordance with the National Institutes of Health guidelines for the humane use of laboratory animals. All experimental procedures involving mice were approved by the Institutional Animal Care and Use Committee of Johns Hopkins University under protocol MO23H131. Johns Hopkins University has received Public Health Service-Approved Animal Welfare Assurance (No. D16-00173) from the National Institutes of Health Office of Laboratory Animal Welfare. Johns Hopkins University is also accredited by the Association for Assessment and Accreditation of Laboratory Animal Care International. Additionally, each mouse was monitored for at least 5 min after administration to ensure a lack of acute toxicity. **Supplementary Figure S2** shows no change in weight gain over time across groups for either vaccine modality.

### Intramuscular vaccination

**Figure 1:**
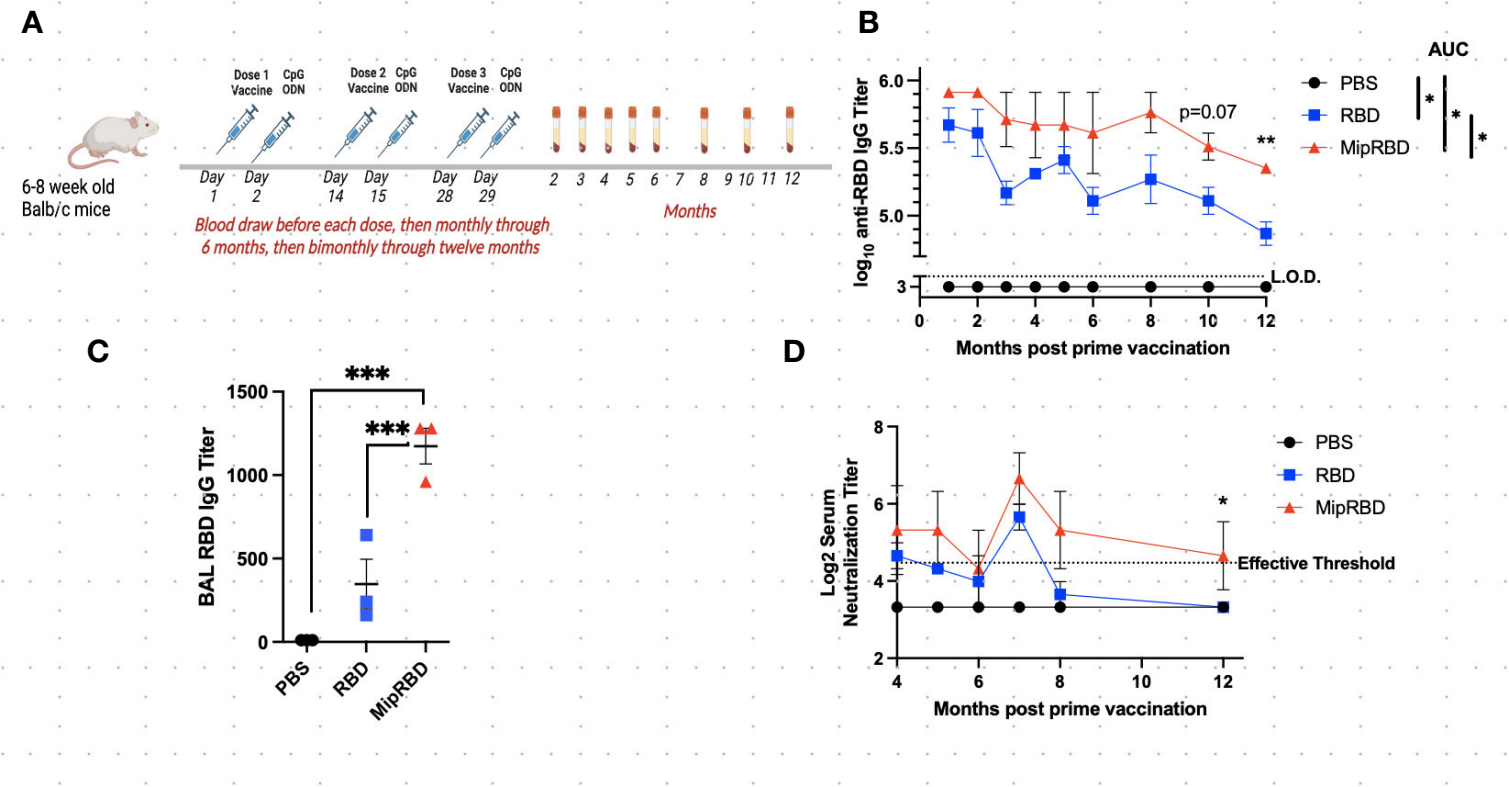
Effect of vaccine formulation on concentration of a specific antibody maintained over 12 months. **(A)** Vaccination and serum-sampling schedule for BALB/c mice, with primary vaccination initiated at 6–8 weeks of age. Mice were immunized IM with 10 μg of plasmid DNA encoding either MipRBD orRBD or with PBS. Immediately following vaccination, each mouse received electroporation, as described in Methods. At 24 h after immunization, each mouse received 50 μg of CpG at the immunization site. Tail vein bleeds were obtained at the indicated time points after the initial immunization. **(B)** Reciprocal antibody titers, determined by ELISA, indicate the highest dilution of serum at which the absorbance was twice the average value of the negative control wells (average of no serum, no secondary antibody, and no substrate wells in duplicate). The antibody titer was log_10_ transformed, and area under the curve (AUC) was conducted to compare differences and significance across the curves’ entireties by nonoverlapping 95% confidence intervals (CI). Symbols represent group means nontransformed. Error bars represent the standard error of the mean (SEM). For AUC, 95% CI were PBS (33), RBD (56.40–58.87), and MipRBD (60.57–63.92). Comparison of MipRBD vs. RBD reciprocal antibody titers at individual time points using multiple unpaired *t*-tests demonstrated a significant difference at the 12-month time point (*p* < 0.05). **(C)** Reciprocal antibody titers in BAL fluid 12 months after the primary immunization, with significance determined by one-way ANOVA. Symbols represent individual mice. **(D)** Reciprocal titers of neutralizing antibodies at different points over the 12 months postprimary immunization. Symbols represent the group means. The significance of differences at individual time points was determined using the Kruskal–Wallis test to compare areas under the curve of the antibody titer dilutions. At the 12-month time point, the RBD and MipRBD differed significantly. Three female mice per group. *p = 0.05; **p < 0.01; ***p < 0.001.

For IM vaccination (**Figure 1**), the DNA vaccine construct was diluted in 1× endotoxin-free PBS, and each mouse received a volume of 50–70 μL by injection into the right gastrocnemius muscle. Immediately following injection, the muscle was pulsed using an ECM 830 Electro Square Porator with 2-Needle Array Electrode (BTX Harvard Apparatus, Holliston, MA, USA) under the following parameters: 106 V, 20 ms pulse length, 200 ms pulse interval, and eight total pulses. CpG type B (ODN1826) (InvivoGen, San Diego, CA, USA) was used as the adjuvant for IM immunization and was diluted in 1× endotoxin-free PBS at 1 mg/mL. The adjuvant was injected IM into the right gastrocnemius muscle at a volume of 50 μL. Three doses of the vaccine were given, each 2 weeks apart, and the adjuvant was given 1 day after every vaccination to allow for the expression of protein from the DNA vaccine. The long-term study utilized a 10-μg dose. The IM immunization used for comparison purposes in the IN immunization studies employed a dose of 50 μg (30).

### Intranasal vaccination

The DNA vaccine construct was diluted in 1× endotoxin-free PBS to a concentration of 2 μg/μL for all groups. Mice were anesthetized before vaccination by inhalation of vaporized isoflurane. For each IN dose, 100 μg of vaccine was administered in each nostril in a volume of 50 μL. The IN vaccine was administered four times at 3-week intervals. IN adjuvant (CpG 25 μg in 50 μL of PBS in each nostril) was administered 1 day after the vaccine and only after the fourth IN vaccination. Each mouse was monitored in an upright position until complete recovery from anesthesia and vaccine absorption was confirmed by lack of nasal discharge.

### Lymphocyte isolation

Under sterile conditions, mouse spleens and lungs were harvested and placed in 1× PBS on ice. Lungs were transferred to wells containing 1 mL of digestion buffer (RPMI media, 167 μg/mL of Liberase, and 100 μg/mL of DNase), minced with scissors, and incubated at 37° for 30 min. Lungs and spleens were ground gently with a pestle over a 70-μM mesh filter into 50-mL conical tubes and immediately centrifuged at 300×*g* for 10 min at 4°. The supernatant was removed, and the pellet was then fully resuspended using 1 mL of ACK lysis buffer (Quality Biological, Gaithersburg, MD, USA) and incubated at room temperature (RT) for 3–4 min. To stop cell lysis, cells were diluted with 20–30 mL of cold 1× PBS and were then pelleted at 300×*g* for 10 min at 4°. After another resuspension in 10 mL of cold 1× PBS and centrifugation under the same conditions, the supernatant was removed and the pellet was resuspended in 1 mL (lungs) or 4 mL (spleens) freezing medium (90% FBS, 10% DMSO) and aliquoted into two (lungs) and four (spleens) tubes for cryostorage using isopropanol cooling containers (Mr. Frosty, ThermoFisher Scientific, Waltham, MA, USA) at −80° for at least 4 h and then moved to −150°.

### Enzyme-linked immunosorbent assay

At the indicated time points postvaccination (**Figure 1A**), approximately 100 μL of mouse blood was collected in either heparin-coated (plasma) or heparin-uncoated (serum) 1.5 mL microcentrifuge tubes by tail vein nicking. Serum samples were allowed to coagulate at room temperature for 0.5–1 h and then spun at 2,200×*g* for 10 min at 4°C. For plasma, samples were spun at 300×*g* for 5 min at 4°C, the supernatant was transferred to a new tube, and the samples were spun at 1,500×*g* for 15 min at 4°C. The serum or plasma supernatant was frozen and stored at −20° until used. Humoral immune responses to RBD protein were measured using enzyme-linked immunosorbent assay (ELISA). The ELISA plates were coated overnight at 4° with 0.2 μg/microwell of S-RBD His-tag recombinant protein (Invitrogen, Rockford, IL, USA) diluted in 100 μL of sterile 1× PBS (2 ng/μL). Each well of the plate was washed three times using 250 μL of washing solution (0.05% Tween 20 diluted in 1× PBS). Plates were emptied, and the residual liquid was discarded before blocking each well with 250 μL of blocking solution (1% BSA in 1× PBS) for 30 min at RT. Following the addition of 100 μL of mouse serum samples serially diluted in triplicate in blocking solution to each well, the plate was incubated at RT for 2 h. After washing the plates six times with 250 μL/well of washing solution, 100 μL of 1:1,000 diluted HRP goat antimouse IgG (H+L) secondary antibody (Biotium, Fremont, CA, USA) was added to each well and incubated at RT for 1 h. The plates were washed six times using the same amount of washing solution again after the secondary antibody incubation, and 100 μL of KPL ABTS^®^ Peroxidase Substrate (SeraCare Life Sciences Inc., Milford, MA, USA) was then added into each well, and the plates were incubated at RT in the dark for 1 h. Data were collected using the Synergy HT at O.D.405 nm (BioTek Instruments Inc., Winooski, VT, USA). Serum samples were diluted across the plate. Antibody titers were calculated as the highest serum dilution that registered absorbance values above (≥) the background threshold. The threshold was defined as twice the average value of control wells. Control wells included in duplicate are (1) all but serum (2), all but secondary antibody, and (3) all but substrate.

### SARS-CoV-2 virus and neutralization assay

SARS-CoV-2 infectious virus titers were determined via a 50% tissue culture infectious dose, as previously described (39). An early SARS-CoV-2 isolate containing the spike D614G mutation was used for all neutralization assays, as previously described (40, 41). All sera were diluted twofold (final dilutions of plasma ranging from 1:20 to 1:2,560), and infectious virus was added at a final concentration of 1 × 10^3^ TCID_50_/mL to the serial dilutions, and incubated for 1 h at room temperature. A 100 μL mixture of virus–serum (containing 100 TCID50 units) was transferred to a 96-well plate of VeroE6-TMPRSS2 cells in sextuplets and then incubated until a cytopathic effect was observed in the controls and the highest sera dilutions. The cells were fixed and stained, and the neutralization titers (NTs) were calculated as the highest serum dilutions that eliminated the cytopathic effect in 50% of the wells (NT50). A positive threshold was defined as NT ≥ 20. An area-under-the-curve analysis then converted the endpoint titer to a continuous variable, and the significance of differences at the different time points was determined using the Kruskal–Wallis test.

### Bronchoalveolar lavage

Wash fluid {1× PBS, 100 µM EDTA from 0.5 M at pH 8 liquid stock (Corning, Glendale, AZ), protease inhibitors (1× PMSF from 200× stock, Cell Signaling Technology, Danvers, MA)} was prepared the day before bronchoalveolar lavage (BAL) and stored at 4°C. Following mouse euthanasia and cardiac puncture, the trachea was exposed, and a mouse endotracheal tube (20 G× 1 in., Kent Scientific Corp., Torrington, CT, USA) was inserted into the trachea to a point just above the carina. The guide needle was removed, and a syringe was attached to the endotracheal tube, which was manually held in place during syringe attachment. Next, 0.5 mL of the wash fluid was slowly injected into the lungs with visual confirmation that the lobes were inflated and that there was no leaking. The injected liquid was aspirated with mild pressure applied to the inflated lobes, as necessary. The aspirated fluid was transferred to a 1.5-mL vial on ice and then transferred to a new tube for centrifugation at 400×*g* for 7 min at 4°C. The supernatant was stored at either 20°C or −80°C, depending on subsequent plans for use.

### Flow cytometry

Cryopreserved cells were recovered by thawing briefly in a water bath at 37° and diluted slowly to 10 mL with warm complete media (RPMI, 10% FBS, 1× antibiotics, 20 mM HEPES, 1% sodium pyruvate, 1% nonessential amino acids, and 1% l-glutamine). Cells were spun for 7 min at 250×*g* at RT and then resuspended in a smaller volume of warm media to obtain a final concentration of 5 × 10^5^–1 × 10^6^ viable cells/well in 200 μL of complete media. The cells were then rested in a 5%CO_2_ incubator at 37° for 2–3 h prior to stimulation with 1 μg of SARS-CoV-2 S protein RBD (Invitrogen, Rockford, IL, USA) at 37° for 16 h. During the final four hours, the cells were incubated with Brefeldin-A at 1× dilution (5 μg/mL; 1 μg in the well) and costimulatory antibodies anti-CD28 and anti-CD49d at 1 μg each per well (BioLegend Cat. No. 420601, No. 102116, and No. 103629). Full-Minus-One (FMO) and positive control wells were stimulated for 4 h with Cell Activation Cocktail (with Brefeldin A) according to manufacturer protocol (BioLegend Cat. No. 423303). After stimulation, 20 μL of EDTA (20 mM of EDTA diluted in 1× PBS at pH 7.4) was added to each well to ensure cells were in suspension and then transferred to a 96-well V-bottom plate. After centrifugation at 300×*g* for 5 min at RT, cells were washed with 150 μL of FACS buffer (0.5% bovine serum albumin (Sigma-Aldrich, St. Louis, MO, USA) in 1× sterile PBS) and pelleted again. Cells were stained with 100 μL/well of live/dead (L/D) stain (1:1,000 dilution in 1× sterile PBS) for 30 min at RT in the dark (LIVE/DEAD Fixable Near-IR Dead Cell Stain Kit, Thermo Fisher Scientific). Cells were pelleted and resuspended in 150 μL of FACS buffer and washed. Following the L/D stain, 50 μL of 2% Fc block (TruStain FcX, BioLegend Cat. No. 101320) was added to each well and incubated in the dark for 15 min on ice. After centrifugation, cells were stained in the dark for 20 min with an antimouse monoclonal-antibody (mAb) cocktail (50 μL per well, diluted in FACS buffer), including 1:500 FITC-conjugated anti-CD4 (BioLegend Cat. No. 100405), 1:200 PercPCy5.5-conjugated anti-CD3 (BioLegend Cat. No. 100217), and 1:200 Alexa Fluor 700-conjugated anti-CD8 (BioLegend Cat. No. 155022). After centrifugation and resuspension in 50 μL of Fixation buffer (Cyto-Fast Fix/Perm Buffer Set, BioLegend Cat No. 426803), cells were then incubated in the dark either at RT for 30 min or at 4° overnight.

For intracellular cytokine staining, an intracellular antimouse mAb cocktail (50 μL per well, diluted in 1× Cyto-Fast Perm buffer) was used to stain the cells in the dark at RT for 20 min. The cocktail includes 1:500 PECy7-conjugated anti-TNF-α (BioLegend Cat. No. 506323) and 1:100 APC-conjugated anti-IFN-γ (BioLegend Cat. No. 505809). Each well received 100 μL of Cyto-Fast Perm buffer for centrifugation at 400×*g* at RT for 5 min. Cells were then washed and resuspended with 150 μL of FACS buffer and read on the Attune™ NxT flow cytometer (Thermo Fisher Scientific, Waltham, MA, USA). Flow data were analyzed using Flow Jo software (FlowJo 10.8.1, LLC, Ashland, OR, USA). FMO control stains utilizing positive control stimulations were used to guide the gating structure. **Supplementary Figures S3, S4** provides the gating structure utilized for analyses as well as representative plots of the data presented in the Results section.

### Statistics

Antibody titers were log transformed for graphical purposes. All statistics were performed on raw, nontransformed data. One-way analysis of variance with Tukey correction was used for comparisons of multiple groups for flow analyses and BAL antibody titers, and multiple unpaired *t*-tests and area-under-the-curve analyses were used to test for the significance of differences in the long-term antibody studies. For neutralizing antibody studies, the areas under the curves of serum dilutions at a given time point were compared using the Kruskal–Wallis test. All experiments shown contained three to six mice per group, as noted. Females are represented by solid color symbols, and males are represented by open symbols. In longitudinal studies, each symbol represents the group mean, and in all other graphs, each symbol represents one mouse. All error bars represent the estimation of the standard error of the mean (SEM). For all tests, *p* ≤ 0.05 was considered to be significant. Prism Graphpad 9 and 10 (San Diego, CA, USA) were used for all statistical analyses and figure generation.

## Results

### Effect of fusion of vaccine antigen with MIP-3α on sustaining IgG and neutralizing antibody concentrations

To evaluate the maintenance of antibody concentrations following vaccination, 6–8-week-old BALB/c mice were vaccinated IM three times at 2-week intervals with saline or with 10 μg of plasmid DNA encoding either codon-optimized MIP-3α-RBD or codon-optimized RBD using the electroporation procedure described in Methods. One day following plasmid inoculation, 50 μg of the adjuvant CpG was injected into the immunization site. Blood for serum IgG or neutralizing antibody concentrations was obtained before each vaccination, 2 weeks after the final vaccination, at monthly intervals through 6 months postvaccination, and at bimonthly intervals thereafter through 12 months (**Figure 1A**). Antibody titers at all time points evaluated were greater in recipients of the fusion vaccine compared to recipients of vaccine encoding only RBD (mean difference = 2.77 + 0.9-fold), with the difference assuming significance at the later time points postvaccination (*p* = 0.07 at 10 months, *p* = 0.007 at 12 months, **Figure 1B**). The area-under-the-curve-analysis of the antibody concentrations over time for the two vaccination protocols also showed nonoverlapping 95% confidence intervals for RBD (57.64; 56.40–58.87) and for MIP-3α-RBD (62.25; 60.57–63.92). Compared to IM immunization with the RBD construct alone, IM immunization with the MIP-3α-RBD vaccine construct yielded significantly higher IgG concentrations in BAL 12 months after the initiation of vaccination (**Figure 1C**, *p* < 0.001). The higher serum IgG antibody concentrations observed at the later time points assumed particular importance, as this resulted in the maintenance at the 12-month time point of neutralizing antibody titers at a critical efficacy threshold (42) (**Figure 1D**). The area-under-the-curve analysis of the antibody titer curves at the 12-month time point indicated a significant difference between recipients of the MIP-3α-RBD vaccine compared to those receiving the vaccine encoding RBD alone (*p* = 0.05).

### Impact of the IN vaccination route and use of the fusion vaccine on the recruitment of T cells to the lungs

**Figure 2:**
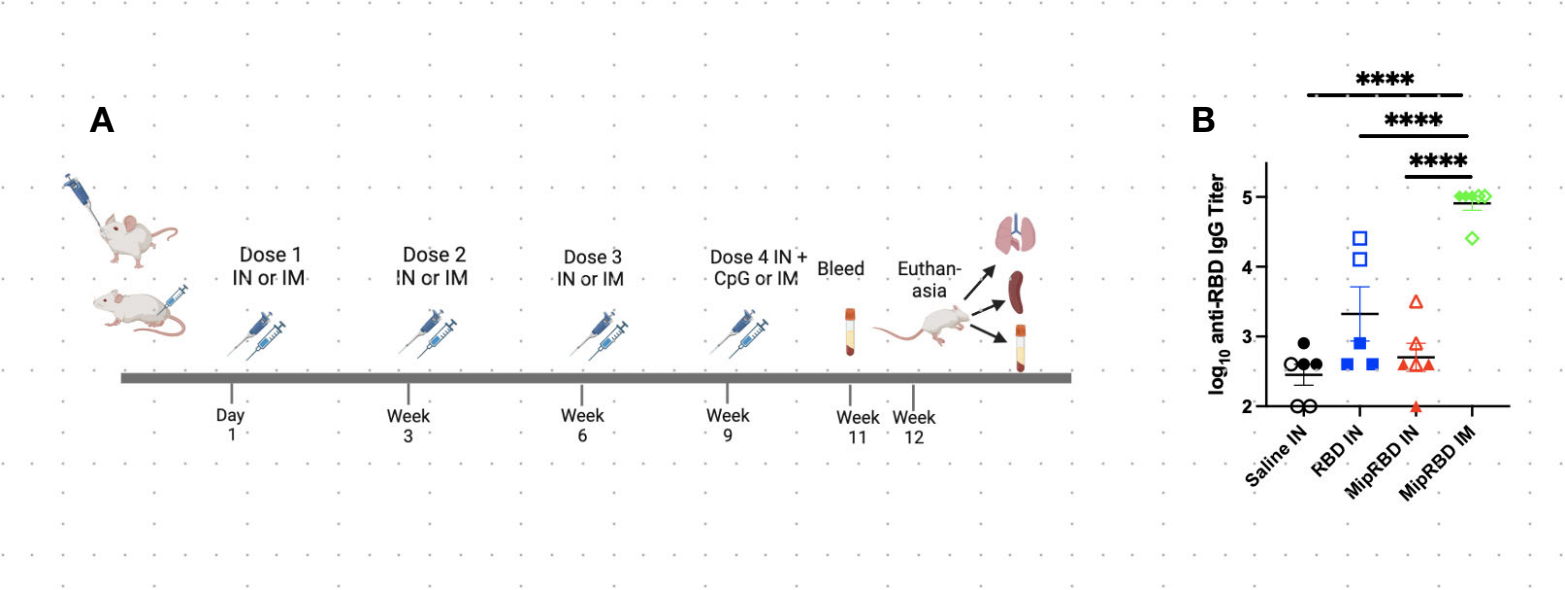
Effect of route of administration and vaccine formulation on serum antibody concentrations. **(A)** Vaccination and serum or tissue sampling schedule for 6–8-week-old BALB/c mice immunized with 50 μg of MipRBD IM followed by electroporation and CpG administration, as described in **Figure 1B**, or IN with 100 μg of plasmid DNA encoding either MipRBD or RBD in a volume of 50 μL of PBS in each nostril. In each nostril, 50 μL of PBS alone was used as a control IN immunization. Electroporation was not employed for IN immunization, and 50 μg of CpG (25 μg in each nostril in 50 μL of PBS) was administered once 1 day after the final immunization. **(B)** Serum antibody titers were determined by ELISA, as described in **Figure 1B**. The significance of differences in titer was determined using one-way ANOVA. Six mice per group, three males (open) and three females (filled), with each symbol representing one mouse. ^****^ *p* < 0.0001.

Because of the potentially important role of T-cell responses in attenuating the severity of SARS-CoV-2 disease, we examined the impact of the route of administration, as well as the role of MIP-3α fusion, in eliciting lung T-cell responses as well as serum antibody responses. These studies evaluated immunogenicity within a shorter time frame and did not analyze the duration of the observed immune responses (**Figure 2A**). The IN vaccination regimen employed was identical to that successfully employed in our previously described TB vaccine formulation (22), in which higher DNA plasmid doses were used to compensate for the lack of electroporation. Mice were immunized on four occasions at 3-week intervals and received either IN immunization with 200 μg of vaccine (100 μg in each nostril in 50 μL of PBS) or IM immunization with 50 μg of vaccine administered with electroporation and use of the CpG adjuvant, as described. For the first three IN immunizations, the plasmid DNA vaccine was administered without adjuvant or electroporation. Because interim antibody analyses indicated no serum antibody response after three IN immunizations, a fourth round of immunizations was undertaken, using the CpG adjuvant (25 μg in 50 μL of PBS in each nostril) for IN, as well as for IM, vaccination. The addition of CpG to the IN vaccine failed to elicit a significant serum antibody response (**Figure 2B**). As indicated (**Figure 2B**), despite receiving four immunizations, serum IgG concentrations remained significantly below those observed with a temporally identical IM immunization protocol (*p* < 0.0001). The MIP-3α fusion vaccine offered no advantage in eliciting systemic antibody responses following IN immunization. Similarly, IN immunization failed to elicit serum IgA antibody responses (data not shown).

**Figure 3:**
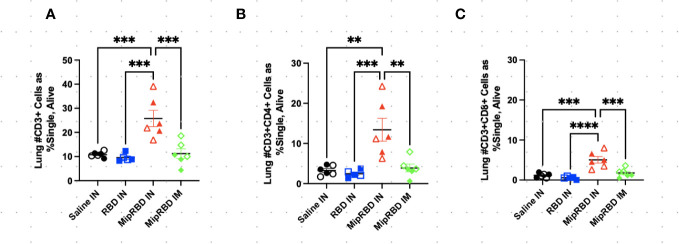
Effect of route of administration and vaccine formulation on T-cell recruitment to the lungs. The vaccination and tissue-harvesting schedule is described in **Figure 2A**. Three weeks after the final vaccination, the recruitment of T-cell subpopulations to the lungs elicited by the different immunization protocols was determined by flow cytometric analysis, as described in Methods. **(A)** All CD3^+^ cells, as well as **(B)** CD3^+^CD8^+^ and **(C)** CD3^+^CD4^+^ cells, were analyzed by normalized cell counts. Differences between groups were analyzed using one-way ANOVA. Six mice per group, three males (open) and three females (filled), with each symbol representing one mouse. ^**^ *p* < 0.01; ^***^ *p* < 0.001; ^****^ *p* < 0.0001.

The inclusion of MIP-3α in the vaccine formulation did, however, have a highly significant effect on the ability of IN immunization to recruit T cells to the lung (**Figure 3A**). At 12 weeks after initial vaccine administration (3 weeks after final vaccination), recipients of the MIP-3α fusion vaccine had an approximately fourfold increase in the number of CD4^+^ T cells (**Figure 3B**) and CD8^+^ T cells (**Figure 3C**) in the lungs compared to recipients of saline, RBD-only vaccine, or, importantly, IM immunization with the MIP-3α-fusion vaccine followed by electroporation and use of the CpG adjuvant. In fact, IM immunization or IN immunization with the RBD alone construct did not elicit T-cell responses in the lung that exceeded those of control mice receiving only PBS.

**Figure 4:**
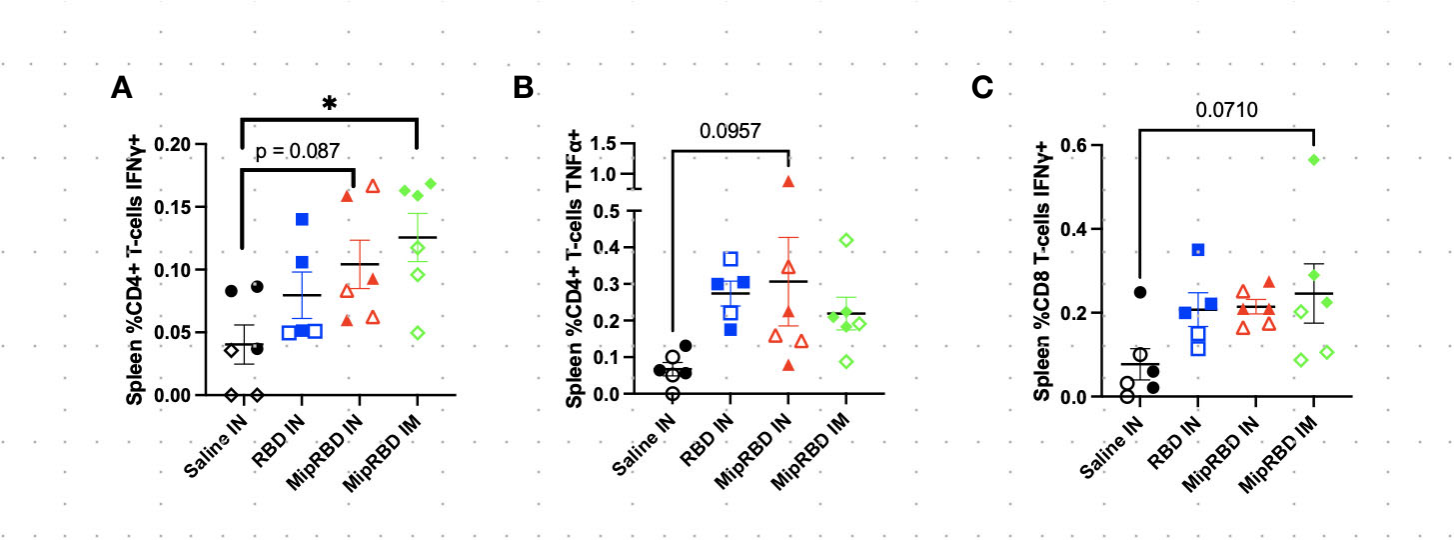
Effect of route of administration and vaccine formulation on IFN-γ and TNF-α responses by T cells in the spleen. The vaccination and tissue-harvesting schedule is described in **Figure 2A**. Harvested lymphocytes were incubated *in vitro* with 1 μg of RBD protein for 16 h, with the final 4 h accumulating intracellular cytokines. Flow cytometric analysis was then performed to evaluate T-cell IFN-γ and TNF-α expression associated with different immunization regimens in **(A)** %CD3^+^CD4^+^ T cells expressing IFN-γ; **(B)** %CD3^+^CD4^+^ T cells expressing TNF-α; and **(C)** %CD3^+^CD8^+^ T cells expressing IFN-γ. Six mice per group, three males (open) and three females (filled), with each symbol representing one mouse. ^*^ *p* < 0.05. Trends of *p* < 0.1 were noted on the graph.

**Figure 5:**
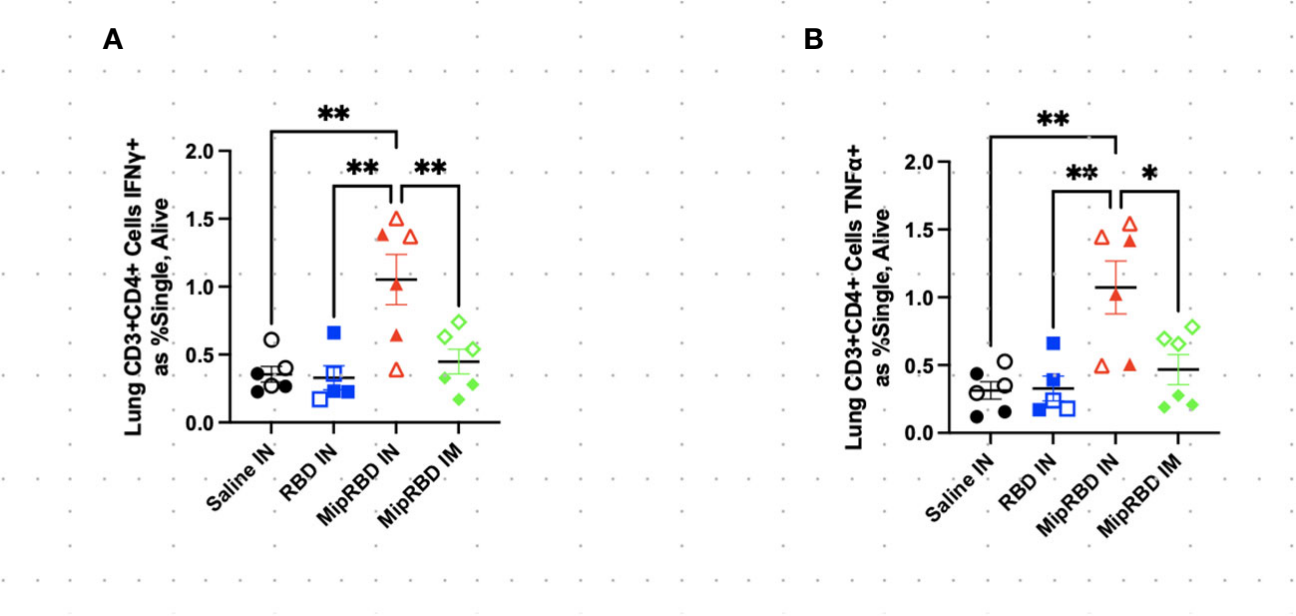
Effect of route of administration and vaccine formulation on IFN-γ and TNF-α responses by T cells in the lung. The vaccination and tissue-harvesting schedule is described in **Figure 2A**. Harvested lymphocytes were incubated *in vitro* with 1 μg of RBD protein for 16 h, with the final 4 h accumulating intracellular cytokines. Flow cytometric analysis was then performed to evaluate T-cell IFN-γ and TNF-α expression associated with different immunization regimens in **(A)** CD3^+^CD4^+^ T cells expressing IFN-γ as a percent of all cells and **(B)** CD3^+^CD4^+^ T cells expressing TNF-α as a percent of all cells. Six mice per group, three males (open) and three females (filled), with each symbol representing one mouse. ^*^ *p* < 0.05; ^**^ *p* < 0.01.

To examine antigen-specific T-cell responses elicited in the spleen (**Figure 4**) and lungs (**Figure 5**) after IM or IN immunization, we evaluated by flow cytometry at the 12-week time point the CD4^+^ and CD8^+^ T-cell expression patterns of activation cytokines IFN-γ and TNF-α following *ex vivo* stimulation with RBD. In the spleen, we see that IM administration of MIP-3α-RBD provides significant IFN-γ expression in CD4^+^ T cells and trending levels in CD8^+^ T cells. Meanwhile, the IN administration of MIP-3α-RBD showed trends of increased IFN-γ and TNF-α in CD4^+^ T cells. At this sample size, RBD without fusion administered IN did not show statistical significance or trends in the spleen. TNF-α levels in CD8^+^ T-cells were too low to provide robust data. Lung stimulation data showed a different picture than the spleen. In a pattern similar to that observed with T-cell recruitment to the lungs, only the MIP-3α fusion vaccine elicited a significantly increased number of IFN-γ- and TNF-α-expressing CD4^+^ T cells post-RBD stimulation in the lungs compared to all other groups (**Figure 5**). The levels of CD8^+^ T cells in the lungs were too low to provide robust data. Overall, the MIP-3α-RBD given IN provides relatively equivalent immunogenicity in the spleen compared to IM but superior immunogenicity in the lungs.

## Discussion

The current preliminary studies have demonstrated that, compared to a DNA vaccine encoding only the SARS-CoV-2 RBD antigen, fusion of the chemokine MIP-3α gene to the RBD antigen gene in the vaccine construct resulted in persistently higher antibody concentrations in serum over a 12-month observation period (**Figures 1B, D**). One limitation of the long-term IM study is the small sample size of three mice per group. Despite this, statistical significance was consistently observed with several parameters over time. Therefore, we hypothesize the results of this preliminary long-term study to be valid. An area-under-the-curve analysis over that time frame indicated that the difference between the total IgG antibody responses to the two vaccine constructs was significant. At the final time point, 12 months following the initiation of vaccination, the concentrations of total IgG antibodies maintained were also significantly different between the two vaccine formulations, indicating the potential for a more extended period of protection against viral infection or severe disease provided by the fusion construct. This extended protection capability was further supported by the finding at the 12-month time point that the concentration of neutralizing antibodies was also significantly higher among recipients of the fusion vaccine, with only the fusion vaccine readily exceeding the threshold considered to be protective (42). Importantly, significantly higher antibody concentrations among recipients of the MIP-3α fusion construct were also observed in BAL at the 12-month time point.

The results also indicate that the fusion of vaccine antigen with chemokine was critical to the recruitment of T cells to the lungs following IN immunization. Upon *in vitro* stimulation with the RBD protein, only mice immunized IN with the MIP-3α fusion vaccine construct elicited IFN-γ^+^ and TNF-α T-cell responses that were significantly above control levels observed in unimmunized mice. IM immunization with the fusion vaccine construct failed to elicit a similar T-cell response in the lungs. The IN immunization experiment contained three mice of each sex per group. However, at this sample size, there were no clear trends of immunological differences between male and female mice.

Previous studies have indicated that, when the species-appropriate MIP-3α fusion product is used, no immune response is generated to the autologous MIP-3α component, even while markedly elevated responses are observed to the targeted antigen (32). While fusing vaccine antigens to DC-targeting ligands is not novel *per se*, studies using that approach have typically employed ligands for receptors found only on mature DC (25–28, 43), forgoing the opportunity to enhance antigen uptake in iDC and modify antigen processing during the earliest stages of the adaptive immune response.

Particularly relevant to the current work, studies by others examining IN immunization determined that MIP-3α played a unique role in eliciting immune responses at that site (44–46). Qin et al. demonstrated that MIP-3α drove DC recruitment to the nasal mucosa and further promoted the development of transepithelial dendrites in these cells. This effect resulted in enhanced antigen uptake and the rapid migration of DC into the draining cervical lymph nodes (46).

Our studies indicate that both the inclusion of MIP-3α in the vaccine construct and IN immunization were critical in the current model system for eliciting effector T-cell responses in the lungs. Multiple studies in the clinical setting have indicated that currently employed IM immunization regimens have not been effective in reducing nasal shedding of viruses in the setting of breakthrough infections [reviewed by Brussow (47)], and studies in nonhuman primates have indicated the importance of T-cell immunity in preventing nasal shedding (13). Le Nouen et al. (48) demonstrated in nonhuman primates the ability of intratracheal/IN immunization with a parainfluenza virus-vectored prefusion stabilized spike protein vaccine to elicit protective T-cell and antibody responses that included prevention of viral shedding in the upper and lower airways. They noted the dependence on T-cell immunity for the prevention of viral shedding. Lei et al. (49) demonstrated the ability of IN immunization with an experimental polyethyleneimine-adjuvanted SARS-CoV-2 spike protein to sustain systemic and BAL antibody levels, as well as lung T-cell responses. Potential disadvantages of these approaches are the reduced likelihood that a viral-vectored vaccine could be used for booster immunizations, the production issues associated with protein vaccines, and the untested clinical safety and efficacy associated with polyethyleneimine used as a vaccine adjuvant.

The current studies identify a novel approach for potentially eliciting both more durable antibody-mediated protection as well as enhancing T-cell responses within the lungs. Both of these outcomes offer the potential for more effective protection against emerging viral variants and for a reduction in viral shedding by vaccinated individuals with breakthrough infections. The results also provide an experimental system in which the individual protective contributions of humoral and cell-mediated immunity can be studied. While the current vaccine was studied as an easily constructed DNA formulation, the conclusions on the role of MIP-3α on the observed responses should be applicable across formulations that avoid issues around the use of viral-vectored or protein vaccines. Future studies can confirm that updated forms of this fusion vaccine will have similar efficacy to new SARS-CoV-2 variants of concern. The speed at which the vaccine could be updated is a strength in the constantly changing viral landscape. Our results suggest that a dual vaccination approach for this construct, IN and IM, would be most effective in providing optimal protection, warranting further study. While DNA vaccines have historically elicited poor responses in human studies, recent clinical studies demonstrating the efficacy of a DNA-formulated SARS-CoV-2 vaccine indicate the potential utility of stable, properly formulated DNA vaccines in the clinical setting (50, 51). These studies represent a preliminary analysis of an approach that will require currently planned future studies that define the duration of IN immunization-elicited T-cell-mediated protection, the roles of different T-cell subsets, the efficacy of this vaccine construct in murine and nonhuman primate challenge models, and the ability of mRNA formulations of this vaccine construct to elicit similar immune responses.

## Data availability statement

The original contributions presented in the study are included in the article/**Supplementary Material**. Further inquiries can be directed to the corresponding author.

## Ethics statement

The animal study was approved by The Animal Care and Use Committee of Johns Hopkins University. The study was conducted in accordance with the local legislation and institutional requirements.

## Author contributions

JG: Conceptualization, Methodology, Supervision, Validation, Writing – review & editing, Formal analysis, Investigation. YH: Formal analysis, Investigation, Methodology, Writing – original draft, Writing – review & editing. CS: Investigation, Methodology, Writing – review & editing. TW: Investigation, Methodology, Writing – review & editing. FC: Investigation, Writing – review & editing. KF: Investigation, Writing – review & editing. JM: Writing – review & editing. YL: Writing – review & editing. AT: Writing – review & editing. RB: Writing – review & editing. PK: Writing – review & editing, Conceptualization, Formal analysis, Funding acquisition. AP: Data curation, Supervision, Writing – review & editing, Formal analysis, Investigation. JS: Investigation, Methodology, Writing – review & editing. ML: Investigation, Methodology, Writing – review & editing. SK: Investigation, Methodology, Writing – review & editing. RM: Conceptualization, Data curation, Funding acquisition, Methodology, Project administration, Supervision, Validation, Writing – original draft, Writing – review & editing.

## Supplementary material

Supplementary Figure 1 Plasmid Design and Construct Verification. **(A, B)** Map of the constructs within the pSecTag2b mammalian expression plasmid designed with Snap Gene software, with full length human Mip-3α fused to the receptor binding domain (RBD) of the Wuhan-Hu-1 isolate of SARS-CoV-2 **(A)** or with the RBD domain only **(B, C)** Single and Double digests of vaccine plasmid as further verification of construct purity and correctness. Each lane represents the digestion product of 500ng of purified DNA plasmid with specified enzymes as run on a 1% Agarose Gel embedded with ethidium bromide at 150V for 20 minutes and visualized by UV light. Relevant band sizes are labeled. **(D)** Expression of protein in mammalian HEK293T cells was verified by Western Blot against the C-myc tag in both cell lysate and supernatant.

Supplementary Figure 2 Mouse Mass Over Time. The percent increase of body mass was calculated over the time course of the IM vaccination series **(A)** and the IN vaccination series **(B)**. By Mixed Effects Models and Area Under the Curve analyses, no significant differences were found across groups.

Supplementary Figure 3 Spleen Gating Strategies and Representative Plots. **(A)** Gating strategy for splenocyte analysis. **(B)** Representative plots for cytokine expression in CD4+ and CD8+ T cells in the spleen. Numbers on plots are percent of parent gate.

Supplementary Figure 4 Lung Gating Strategies and Representative Plots. **(A)** Gating strategy for lung analysis. **(B)** Representative plots for lung T-cell infiltration. **(C)** Representative plots for cytokine expression in lung T cells. Numbers on plots are percent of parent for panel A and percent of Alive gate for panels B and C.
