# Supplementary figures and images for: A SARS-CoV-2 RBD vaccine fused to the chemokine MIP-3α elicits sustained murine antibody responses over 12 months and enhanced lung T-cell responses
Source: Front Immunol. 2024 Feb 2;15:1292059. doi: 10.3389/fimmu.2024.1292059 (PMC10870766; doi:10.3389/fimmu.2024.1292059)

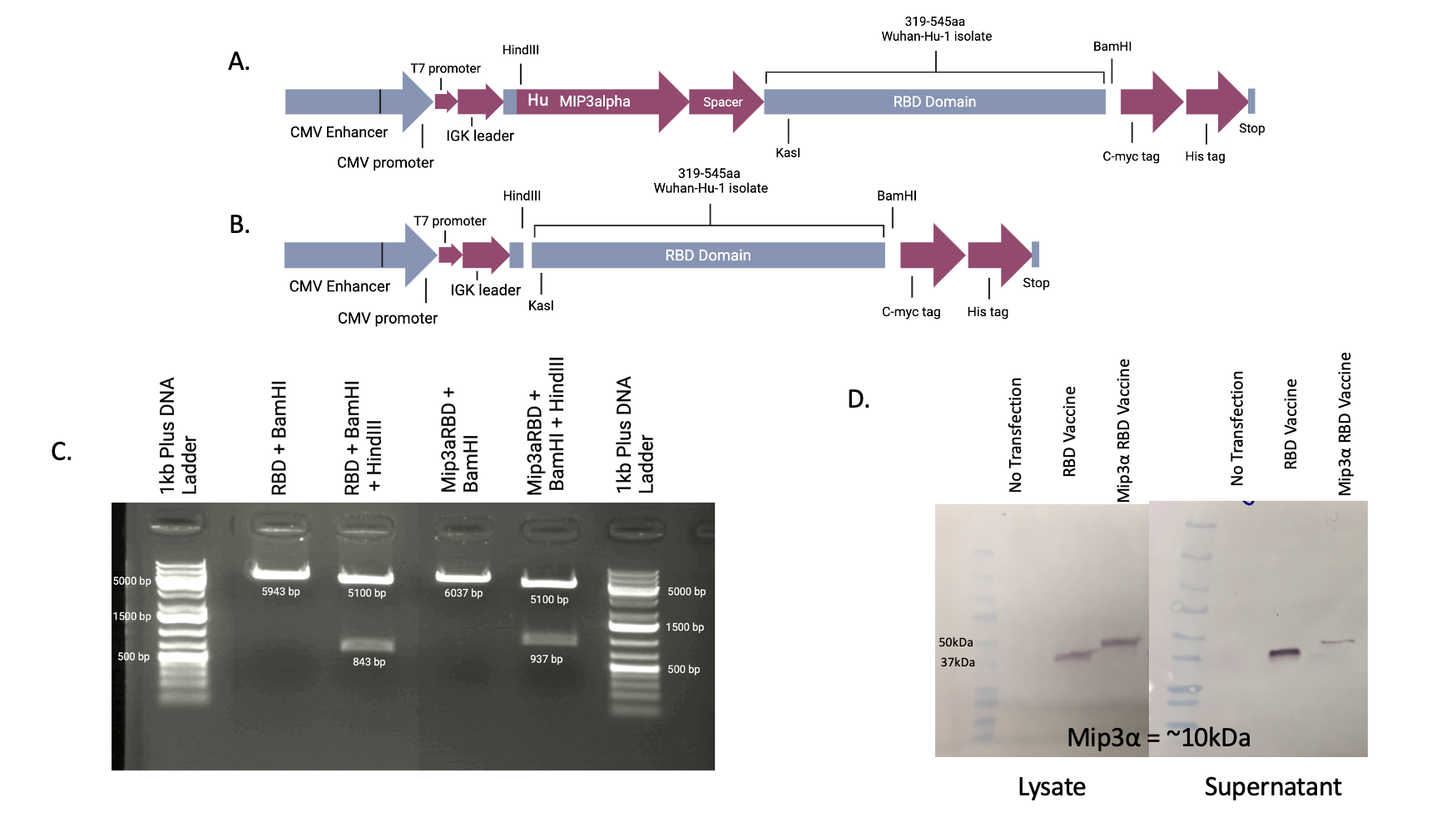

Supplement: Supplementary Figure 1 — Plasmid Design and Construct Verification. (A, B) Map of the constructs within the pSecTag2b mammalian expression plasmid designed with Snap Gene software, with full length human Mip-3α fused to the receptor binding domain (RBD) of the Wuhan-Hu-1 isolate of SARS-CoV-2 (A) or with the RBD domain only (B, C) Single and Double digests of vaccine plasmid as further verification of construct purity and correctness. Each lane represents the digestion product of 500ng of purified DNA plasmid with specified enzymes as run on a 1% Agarose Gel embedded with ethidium bromide at 150V for 20 minutes and visualized by UV light. Relevant band sizes are labeled. (D) Expression of protein in mammalian HEK293T cells was verified by Western Blot against the C-myc tag in both cell lysate and supernatant. [file Image_1.tiff]

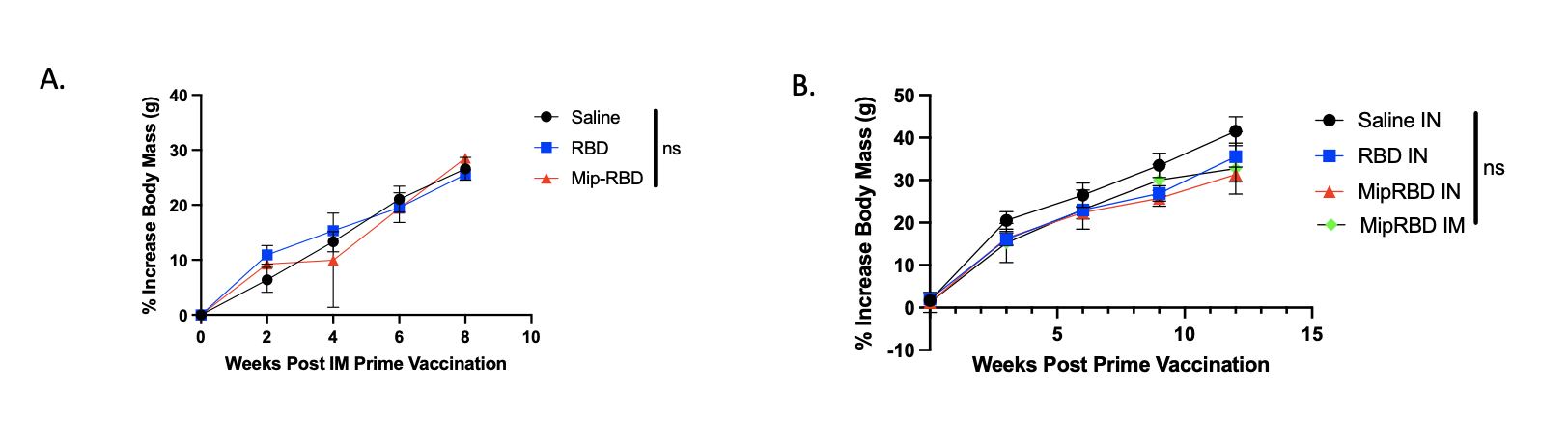

Supplement: Supplementary Figure 2 — Mouse Mass Over Time. The percent increase of body mass was calculated over the time course of the IM vaccination series (A) and the IN vaccination series (B). By Mixed Effects Models and Area Under the Curve analyses, no significant differences were found across groups. [file Image_2.tiff]

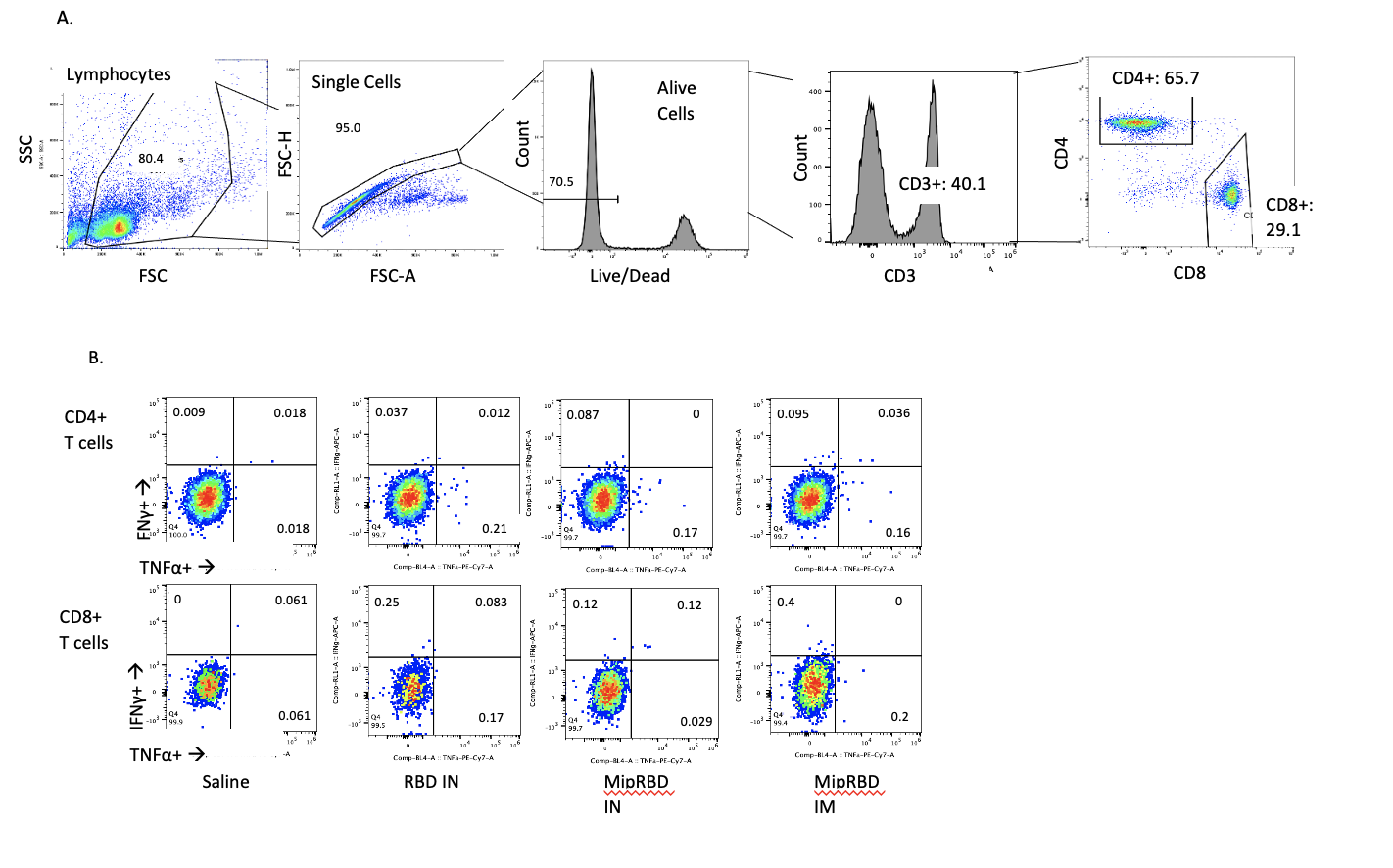

Supplement: Supplementary Figure 3 — Spleen Gating Strategies and Representative Plots. (A) Gating strategy for splenocyte analysis. (B) Representative plots for cytokine expression in CD4+ and CD8+ T cells in the spleen. Numbers on plots are percent of parent gate. [file Image_3.tiff]

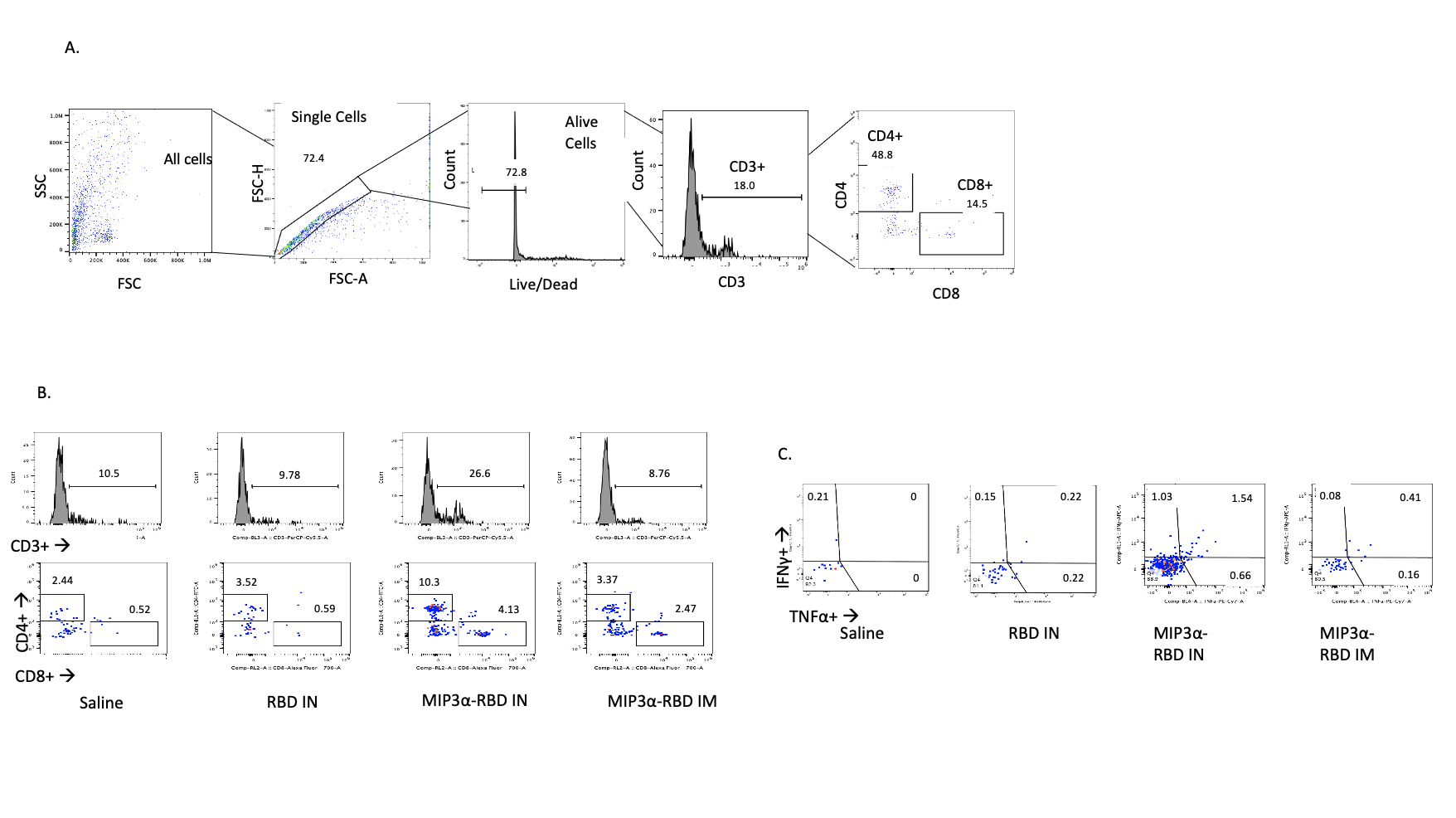

Supplement: Supplementary Figure 4 — Lung Gating Strategies and Representative Plots. (A) Gating strategy for lung analysis. (B) Representative plots for lung T-cell infiltration. (C) Representative plots for cytokine expression in lung T cells. Numbers on plots are percent of parent for panel A and percent of Alive gate for panels B and C. [file Image_4.tiff]
